# Factitious schizophrenia

**DOI:** 10.4103/0019-5545.55944

**Published:** 2005

**Authors:** Sandeep Grover, Suresh Kumar, Surendra Kumar Mattoo, Nitesh Prakash Painuly, Gaurav Bhateja, Rajinder Kaur

**Affiliations:** * Senior Resident, Department of Psychiatry, Postgraduate Institute of Medical Education and Research (PGIMER), Chandigarh; ** Assistant Professor, Department of Psychiatry, Postgraduate Institute of Medical Education and Research (PGIMER), Chandigarh; *** Additional Professor, Department of Psychiatry, Postgraduate Institute of Medical Education and Research (PGIMER), Chandigarh; **** Senior Resident, Department of Psychiatry, Postgraduate Institute of Medical Education and Research (PGIMER), Chandigarh; ***** Junior Resident, Department of Psychiatry, Postgraduate Institute of Medical Education and Research (PGIMER), Chandigarh; ****** Clinical Psychologist, Department of Psychiatry, Postgraduate Institute of Medical Education and Research (PGIMER), Chandigarh

**Keywords:** Schizophrenia, factitious disorder

## Abstract

Factitious disorder is a challenging phenomenon in clinical practice. An inconsistent clinical picture and the presence of symptoms that do not respond to the seemingly appropriate treatment should alert the clinician about the possibility of such a diagnosis. A case of factitious psychological symptoms suggestive of schizophrenia is reported, and the issues of diagnosis and management are discussed in the light of the available literature.

## INTRODUCTION

Factitious disorder (FD) is a unique and challenging phenomenon in clinical practice. It has been known for many centuries and has been mentioned in both the professional and lay literature. It involves efforts to garner gratification intrinsic to the sick role through the simulation, exaggeration, aggravation or induction of physical or psychological signs and symptoms.^1^ In this role, sick individuals are excused from social responsibility, are expected to perceive their condition as undesirable, lack voluntary control over the disease and are therefore not considered at fault, and are expected to seek help in ameliorating or curing the condition.^2^ Usually, the patient's primary goal is to receive medical, surgical, or psychiatric care to gratify some unconscious psychological needs.^1^ Asher^3^ used the term ‘Munchausen syndrome’ for the first time to describe the category of patients who chronically fabricate symptoms to gain admission to hospital.

Factitious disorders in which physical symptoms and signs are induced have been frequently described, in the form of factitious haematuria, haemoptysis, torsion dystonia, faeculent urine, self-mutilation, pulmonary manifestations, dermatitis artefacta, fever of unknown origin, renal stones, hypo-glycaemia, diarrhoea and anaemia. In neurological practice, seizures and paralysis are probably the most commonly feigned diseases.^4^

Factitious disorders with psychological presentation are most frequently seen in conjunction with physical complaints. Where FDs consists exclusively of psychological symptoms, the individual exhibits peregrination, antisocial behaviour, and lack of intimate and sustained relationships.^1^ FDs with symptoms of depression,^5^ bereavement,^6^ post-traumatic stress disorder^7^ and alcohol dependence^8^ have been described in the past. There are only isolated case reports and case series of feigned psychosis. Ritson and Forrest^9^ described 12 patients who ‘played schizophrenia’; Cheng and Hummel^10^ described two patients with ‘mental Munchausen syndrome’, one of whom repeatedly simulated an acute psychotic state and Pope *et al*.^11^ described nine patients with factitious psychosis.

There have been no reports of ‘factitious schizophrenia/ psychosis’ from India. A case of factitious schizophrenia is presented and the associated issues are discussed in the light of the available literature.

## THE CASE

An 18-year-old unmarried male, a student of class XII, from an urban nuclear family of middle socioeconomic status was admitted to the hospital following a suicide attempt. His physical examination was normal except for the presence of left-sided gynaecomastia. On mental status examination, he expressed ideas of hopelessness and suicidal urges, in addition to commenting and commanding auditory hallucination.

He was first seen in our outpatient services two years earlier and was diagnosed as a case of schizophrenia because of the presence of delusions, and commenting and commanding hallucinations. He described the psychotic symptoms and in fact provided details on various parameters of these phenomena. Once the diagnosis of schizophrenia was confirmed, his parents were psychoeducated about his problem and their role in the treatment. He was prescribed Tab. risperidone up to 4 mg per day; up to 50% improvement in the symptoms was reported. Six months later, when the development of left-sided gynaecomastia forced a shift in treatment to Tab. ziprasidone up to 160 mg/day, he reported worsening of his symptoms. When his father turned down his demand for a computer, he made a suicidal attempt that led to the hospitalization.

Within 2–3 days of admission, he became asymptomatic. For the next 2–3 days, he was reluctant to give interviews and insisted that his father should not be around while he was being interviewed. Over the next one week, on repeated interviews, it was noticed that he gave inconsistent information regarding the duration, frequency and intensity of his symptoms. This made the treating team suspect the diagnosis, and further exploration was done.

His family assessment revealed a strict and authoritarian father, who had anankastic traits. His mother was passive and submissive, and for four years was receiving antidepressants for dysthymia. The four members of the family (the patient's parents and his younger brother) were living in a single room and there was a severe relationship problem between the parents, mainly in the context of a sexual relationship.

Since his early teens, the patient was found to become tense before exams, and become anxious and have anger outbursts over minor issues at home. Since the age of 16 years, he had been visiting a psychiatrist and was being treated for depression with fluoxetine and paroxetine with good compliance but no benefit. At the age of 16, he had transient blindness for 12 hours which improved suddenly after a pill of alprazolam and ophthalmological examination (which did not reveal any abnormality). Since the age of 17 years, when he was seen by a neurologist for an episode of ‘seizure’, he had been receiving sodium valproate.

The IQ of the patient was 77 and over the years there was a decline in his academic performance. In class VIII, the patient's score was 65%, which fell to 45% in class X. In class XII, he had to shift from the commerce stream to the arts stream because of academic difficulties. There was no problem in his social and interpersonal functioning.

Personality assessment by the International Personality Disorder Evaluation (IPDE, WHO)^12^ showed that he had an emotionally unstable personality disorder (impulsive type) with anxious and dependent traits. On psychological testing, he was found to be submissive, low on paranoid tendencies and social desirability, and high on psychoticism; was preoccupied with sex, had conflict with authority, poor reality orientation and conflicts with the male sex. On the Sentence Completion Test^13^ and Thematic Apperception Test,^14^ he was ambitious, preoccupied with his parents' sexual relationship; had feelings of inferiority and impulsivity, had conflict with the father and was dependent on the mother.

Because of the diagnostic doubts, he was taken up for supportive psychotherapy during which he was allowed to ventilate. During the sessions, he revealed that since the age of 11 years, he frequently saw his father forcing sex on his mother and she being irritable the next day. He was greatly distressed by this and had experienced a decline in sleep and academic performance. He also had masturbatory guilt, which he attributed to excessive masturbation due to preoccupation with his father's sexual behaviour. He was very angry with his father and wanted to save his mother from him.

Since the onset of his ‘mental illness’ his father had started sleeping with him resulting in marked decrease in sexual encounters between the parents. Also, the parents in general and the father in particular had started giving him extra care and fulfilling more of his demands.

After the establishment of a stable therapeutic relationship, the patient disclosed that, for the past two years, he had lied to the treating psychiatrists that he had been hearing voices; he confessed that he had falsely agreed to hearing voices on direct questioning by the psychiatrist. Later, he found his parents very much distressed, a marked change took place in their behaviour and they started fulfilling his demands. This change in parental behaviour had encouraged him to continue reporting these symptoms without having any understanding of the implications. He also admitted to having feigned ‘loss of vision’ and ‘seizure’ in the past.

From the psychotherapeutic ventilation sessions, it was apparent that initially, the patient did not have any external motive for the production of symptoms, but later, continued to report them because it helped him to get the attention of his parents. Further, adopting the sick role fulfilled the psychological needs of the patient, i.e. saving his depressed mother from his authoritarian father. This concurrently also fulfilled his dependency needs.

His medications were tapered off and stopped. His diagnosis was revised to factitious disorder (predominantly psychological signs and symptoms) along with an emotionally unstable personality disorder (impulsive type). The patient was taken up for supportive psychotherapy on a weekly basis and has been doing well without any medication for the past two years. He had joined a professional course in law, but later dropped out of this course.

## DISCUSSION

The *Diagnostic and statistical manual of mental disorders,* fourth edition (DSM-IV)^15^ describes three essential features of FD: the intentional production or feigning of physical or psychological signs or symptoms, the motivation for the behaviour is to assume the sick role, and external incentives for the behaviour (such as economic gain, avoiding legal responsibilities, or improving well-being, as in malingering) are absent. In our case, the patient feigned both psychological and physical symptoms (predominantly psychological) and there was no apparent external incentive for such a behaviour.

Aetiologically, FD serves the function of meeting the psychological needs of patients. One of these needs could be to gain a sense of control and mastery over some traumatic childhood illness.^16^ Another common theme is masochism, where patients feel a compulsion to suffer and seek self-punishment as atonement for various forbidden emotions and wishes, such as sexual excitement or anger. A third common psychological factor in FD is that of dependency gratification. Many patients with FD experience significant deprivation in childhood. FD may be a way to have their unfulfilled dependency needs met in a socially sanctioned medical setting. Some authors have also attributed FD to a need to feel superior to authority figures, which is gratified by being able to deceive the therapist. The behaviourist views FD as a coping mechanism learned and reinforced in childhood.^1^,^17^ Our patient can be conceptualized as an adolescent boy with poor coping abilities, who wanted to save his submissive and depressed mother from his authoritarian father by adopting the sick role, which concurrently fulfilled his dependency needs.

It has also been reported that patients with FD usually have a poor sense of identity, poor sexual adjustment, poor frustration tolerance, strong dependency needs and narcissism.^1^ The personality assessment and clinical picture of this patient demonstrated the presence of many of these features. Some studies also report these patients to be mentally subnormal or in the low average range,^4^ as was the case in this patient.

The most important conditions to be ruled out in patients with FD are malingering and an authentic medical/psychiatric illness. Malingering and FD have similar features of conscious, intentional production of signs and symptoms of a disease. Malingering has a goal of achieving some external incentives such as financial compensation, avoidance of military duty, evasion of criminal prosecution, avoiding work, obtaining financial compensation, or obtaining drugs. Patients with malingering are frequently uncooperative for diagnostic evaluation and in complying with the prescribed treatment regimen. The diagnoses is often a problem and the symptoms can be puzzling. A patient might malinger to obtain tangible gains associated with the sick role but might still enjoy the nurturing and care that such a role provides.^18^ In FD, there is no observable external reason for symptom production/ maintenance. The only apparent motivation is assuming the role of a patient. In both FD and conversion disorder, the motivation for symptom production is unconscious. In our case, the patient initially feigned hallucinations without any motivation in the form of an external incentive; however, once his family started giving him extra care, he continued to feign the symptoms without understanding their nature and severity, and also kept on taking medication without resistance. Hence, it can be concluded that the initial motivation for the feigning of symptoms was intrapsychic but later, the feigning might have been continued because of both intrapsychic and materialistic gains. FD differs from conversion disorder because in the latter condition, symptoms are produced unconsciously. Observing patients when they think they are not being observed gives a clue to the diagnosis (e.g. the conversion patient with paralysis will remain paralysed whereas a patient with FD may move their limbs). FD needs to be differentiated from other somatoform disorders such as hypochondriasis, somatization disorder, pain associated with psychological factors, and body dysmorphic disorder.^17^

The feigned psychosis is usually suspected by the nursing staff and junior medical staff, but experienced clinicians are reluctant to consider it.^19^ The following are some clues in the presentation and clinical picture of patients which might help in increasing a physician's level of suspicion to arrive at a diagnosis of FD: (i) a puzzling, unusual and inconsistent clinical picture; (ii) symptoms that do not respond to the seemingly appropriate treatment; (iii) an unusually large amount of previously sought medical care at a relatively young age; (iv) an unusual grasp of medical terminology; and (v) the presence of symptoms or behaviours when the patient is being observed.^17^,^20^ In our case, the patient gave an inconsistent history, was seeking medical treatment for past two years, had not responded to appropriate treatment, and his suicide attempt did not appear to be related to psychosis, rather it appeared to be manipulative behaviour. Unlike malingering, there was no tangible preplanned gain from playing a sick role. It was a later discovery. The initial motivation for reporting psychotic symptoms was not obvious to the patient, i.e. he was ‘unconscious’ of this. The patient was aware that he was feigning but did know the reason why. Here lies the distinction between FD and malingering. The sexual behaviour of the father was reported only later during psychotherapy and the patient had not earlier made the link between the father's sexual behaviour and his feigning of symptoms. Due to these reasons, the patient was considered to have FD. However, malingering cannot be completely ruled out in this case because the patient had an external tangible gain by feigning symptoms in the form of saving his mother from his father's sexual behaviour and getting his materialistic demands fulfilled.

Patients with FD present a challenging treatment problem. Use of supportive, face-saving techniques may help a patient to accept appropriate treatment The most important aspect in the management of these patients is to secure an enduring and stable patient–therapist relationship, which can be achieved by being non-confrontational and reframing the manifestation as a ‘cry for help’.^1^ This approach was followed in our case, and was fruitful. Ritson and Forest^9^ proposed the ‘contract conference’ approach for these patients in which the therapist emphasizes the need for the patient to express him/herself in the common language of difficult relationships, feelings and problems in the living instead of the language of illness. Another important issue in management involves the therapist's own counter-transference reactions. Discussing the same with superiors or colleagues often leads to the avoidance of ineffective management. Predictors of a favourable response to treatment include the presence of an underlying treatable psychiatric condition such as depression, anxiety, substance abuse or conversion symptoms; the absence of borderline or antisocial personality traits; good psychosocial supports; and the ability to form a therapeutic alliance with a therapist, characterized by the capacity to establish and maintain rapport, accept confrontation and comply with treatment recommendations.^1^,^17^ Although our patient has an emotionally unstable personality disorder, impulsive subtype, the most likely reason for the good results was the establishment of a good therapeutic alliance.

There are a few studies on the outcome of feigned psychosis. Pope *et al*^11^ in a 4–7-year follow-up study found that none of their patients went on to develop a typical psychotic disorder. In another follow-up study of 3 months to 10 years' duration, Hay^19^ found that 4 out of 5 of their cases went on to develop true schizophrenic illness and concluded that simulation of schizophrenia is often the prodromal phase of genuine illness, occurring in the background of a markedly abnormal personality. Rogers *et al.*^21^ have also drawn a similar conclusion.
